# The other side of comparative genomics: genes with no orthologs between the cow and other mammalian species

**DOI:** 10.1186/1471-2164-10-604

**Published:** 2009-12-14

**Authors:** Raffaele Mazza, Francesco Strozzi, Andrea Caprera, Paolo Ajmone-Marsan, John L Williams

**Affiliations:** 1 Istituto di Zootecnica, Università Cattolica del Sacro Cuore, via Emilia Parmense 84, 29100 Piacenza, Italy; 2 IDRA Lab, Parco Tecnologico Padano, via Einstein, Loc Cascina Codazza, 26900 Lodi, Italy; 3 CERSA, Parco Tecnologico Padano, via Einstein, Loc Cascina Codazza, 26900 Lodi, Italy

## Abstract

**Background:**

With the rapid growth in the availability of genome sequence data, the automated identification of orthologous genes between species (orthologs) is of fundamental importance to facilitate functional annotation and studies on comparative and evolutionary genomics. Genes with no apparent orthologs between the bovine and human genome may be responsible for major differences between the species, however, such genes are often neglected in functional genomics studies.

**Results:**

A BLAST-based method was exploited to explore the current annotation and orthology predictions in Ensembl. Genes with no orthologs between the two genomes were classified into groups based on alignments, ontology, manual curation and publicly available information. Starting from a high quality and specific set of orthology predictions, as provided by Ensembl, hidden relationship between genes and genomes of different mammalian species were unveiled using a highly sensitive approach, based on sequence similarity and genomic comparison.

**Conclusions:**

The analysis identified 3,801 bovine genes with no orthologs in human and 1010 human genes with no orthologs in cow, among which 411 and 43 genes, respectively, had no match at all in the other species. Most of the apparently non-orthologous genes may potentially have orthologs which were missed in the annotation process, despite having a high percentage of identity, because of differences in gene length and structure. The comparative analysis reported here identified gene variants, new genes and species-specific features and gave an overview of the other side of orthology which may help to improve the annotation of the bovine genome and the knowledge of structural differences between species.

## Background

With the rapid increase in the amount of genome sequence data available, the automated identification of orthologous genes between species becomes of fundamental importance to facilitate functional annotation and for comparative or evolutionary genomics. Homologous proteins descend from a common ancestor and may be classified as either orthologous (orthologs) or paralogous (paralogs)[1]. Orthologs are commonly defined as the functional equivalent genes between species, which may have diverged after a speciation event, whereas genes created by a duplication event, either before species divergence (out-paralogs) or after a speciation event (in-paralogs), are known as paralogs. Orthologs typically retain similar domain architecture and function and such conservation is an important component in comparative analysis as well as in the annotation of proteins of unknown function, in the characterization of gene function, for evolutionary genomics and the identification of conserved regulatory elements. In contrast, paralogs may have diverged significantly and acquired new functions, e.g. through point mutations or recombination between domains [1], even though a recent review by Studer and Robinson-Rechavi [2] proposed a model in which both orthologs and paralogs diverge in proportion to time of the duplication event and therefore functional changes can occur for both.

Complete and precise delineation of protein coding genes in the genome and the process of assigning gene orthology remains a challenging task in mammalian genomes because of their large size, the difficulty of creating accurate gene models, the complexity of protein domain architecture and the high frequency of gene duplication events, that create large gene families. Errors in ortholog predictions can significantly affect downstream analyses; as a result there has been increasing interest in high quality ortholog prediction techniques.

During the last decade, there have been several methods proposed for routine generation of genome-wide orthology descriptions, which rely mainly on phylogeny, homology or integrated approaches. Orthology detection methods, based mainly on phylogeny, and implemented in software such as RIO [3], Orthostrapper [4,5], PhiGs [6], PhyOP [7], TreeFam [8], or based on evolutionary distance (RSD [9,10]), generally do not erroneously report genes as orthologous (false positive, FP), but have a high frequency of missed orthologs (false negative, FN). However, it is difficult to automate phylogenetic analysis approaches for genome-wide analysis, therefore prediction of orthologs for large genome datasets has been typically performed using homology based methods which compare reciprocal-best-BLAST-hits (RBH).

The most frequently used BLAST-based homology methods for detecting orthologous genes include those of BLASTP [11], COG (Cluster of Orthologous Groups [12]), KOG (euKaryotic Orthologous Groups [13]), The Institute for Genomic Research (TIGR) EGO/TOGA database [14], InParanoid/MultiParanoid [15-17], TribeMCL [18], OrthoMCL [19,20], KEGG Orthology [21], Roundup [10], MSOAR [22], the OMA project [23] and HomoloGene [24]. However, homology methods used to infer orthology often have high FP error rates and low FN error rates, such as observed with BLASTP, where orthology "hits" typically include true orthologs but also many false positive results [25], the later including paralogs and members of gene families. Moreover, the BLAST searches often return, as the highest scoring hit, a protein that is not the nearest phylogenetic neighbour of the query sequence [26]. In summary, phylogeny-based methods are characterized by high specificity and BLAST-based methods by high sensitivity.

To bypass the limits of single phylogeny or homology methods, in this work we used the Ensembl orthology prediction pipeline as reference [27]. Ensembl uses an integrated approach starting from a homology-based method which builds gene-models using species-specific known sequences and proteins from other species aligned to the target genome. All annotated transcripts are based on experimental evidence and the automated pipelines rely on the mRNAs, ESTs and protein sequences submitted into public databases by the scientific community. Therefore, Ensembl does not annotate genes for which there is no prior evidence of a transcribed sequence. Next, the gene orthology and paralogy predictions are generated using a bioinformatic pipeline where maximum likelihood phylogenetic gene trees (generated by TreeBeST) play a key role. This method produces trees that are the most consistent with the conservation of synteny between species and gives fewer anomalous topologies than single protein-based phylogenetic methods [28]. The Ensembl method does not provide a complete gene set for each species, as it has been demonstrated by genome annotation with different methods that have been able to identify many genes in addition to those annotated by the Ensembl automated pipeline, e.g. 700 chicken genes in addition to the Ensembl gene list [29] and more than 1,000 additional genes between mouse and human genome [30].

The current cattle genome sequence (Btau version 4.0) was based on about 7× genome coverage with 90% of the total sequence placed on the 29 autosomes and X chromosome. This last release was assembled by creating sequence contigs arranged into scaffolds, on the basis of sequence overlap and BAC ends contig data. The scaffolds were then placed on chromosomes and ordered using BAC and radiation hybrid physical maps. Most sequence contigs remain unchanged from the previous release (Btau3.1), but scaffold assembly was improved. Automated annotation identified about 22,000 genes, with a core set of 14,345 orthologs found between cattle and seven mammalian species [31]. Over 4,000 genes were also manually annotated and orthology prediction with dog, human, mouse, rat, opossum, and platypus genomes was resolved for more than 75% of the predicted bovine genes.

In this work we developed a BLAST-based method to explore the current annotation of the bovine genome and to describe those genes that were classified as being non-orthologous between bovine and other mammalian species (human, mouse and dog), according to Ensembl classification. We used a double comparative approach to identify a set of bovine genes that had no orthologs in the other three mammalian species and a set of orthologs in human, mouse and dog with no bovine counterparts. Within the first set we expected to find genes with species-specific features, assembly and annotation problems in addition to bovine specific genes, the so called orphan genes [32]. These are coding sequences having no matches with genes of other annotated species. The orphan genes arose mainly from duplication events, and recent experimental evidence describes them as fast evolving genes [33,34]. The second set contains genes that appear to be absent from the bovine genome, while being present in the other species. Alignments, ontology, manual curation and publicly available information were exploited to classify and investigate groups of genes. Differences between species and possible species-specific features or problems in the assembly of genomes were investigated in order to explain missing orthologs.

## Results

### *In silico *libraries set up

**Figure 1:**
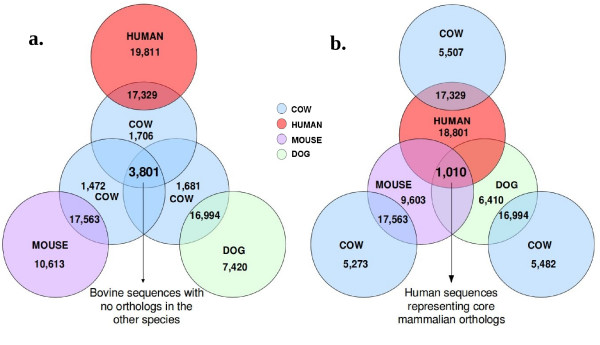
**Venn diagram representation of the results obtained from the queries in Ensembl release 50**. Each colored circle represents a gene set for a specie. a) query result returning 3,801 cow genes with no orthologs in human, mouse and dog. b) query results returning 1,010 human genes representing core mammalian orthologs having no orthologs in cow but with orthologs in mouse and dog.

The full gene sets for cow, human, mouse and dog, were downloaded from Ensembl release 50 to a local database, and comprised 22,836, 36,396, 28,329 and 23,550 genes, respectively. The data used here consisted of genes found to be non-orthologous between cow and human which were further filtered to constitute a core of mammalian orthologs by adding to the comparison the gene sets of mouse and dog, currently the most complete mammalian genomes in terms of sequence information [35,36]. The initial query in Ensembl 50 using the Biomart tool returned 5,507 bovine genes with no orthologs in human and 19,811 human genes with no orthologs in cow. The high number of human non-orthologous genes is explained by the larger number of annotated genes in the human genome. Mouse and dog gene sets, as described in the method section, were then added to the query which retrieved 3,801 bovine genes with no orthologs in human, mouse and dog, and 1,010 human genes with no orthologs in cow but with orthologs in mouse and dog, as represented in the Venn diagram in Figure 1. The two queries are slightly different in principle because the final cow dataset will contain bovine orphan genes, while the human dataset includes genes that can be considered core mammalian orthologs, apparently missing in cow. These two resulting *in silico *data libraries were the subjects for a two-way comparison based on the alignment of the genes from a library of one species to the whole genome sequence of the other species; i.e. bovine genes were aligned to the human genome ("cow *vs*. human comparison") and human genes to the bovine genome ("human *vs*. cow comparison").

### Sequence alignments to genomes

**Figure 2:**
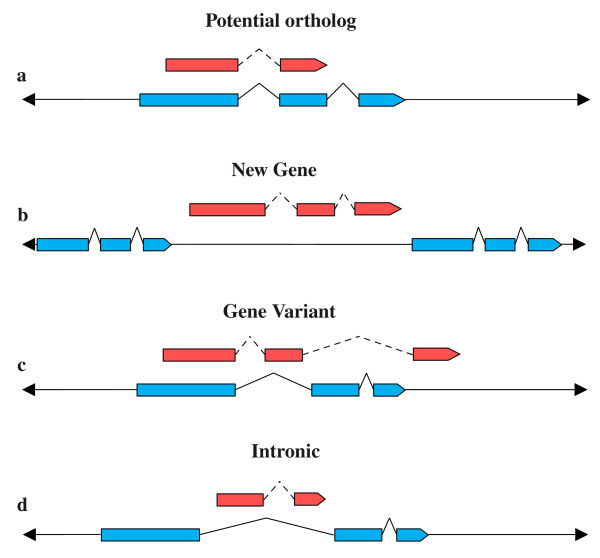
**Schematic representation of the four categories for the aligned genes**. Red boxes indicate the aligned transcript, blue boxes indicate exons of annotated genes on the genome. a) potential ortholog, b) new gene, c) gene variant, d) intronic.

The longest transcript (canonical transcript) for each non-orthologous gene from the starting libraries was aligned to the genome of the other species, and then the canonical transcripts grouped in six distinct categories; four containing the genes that could be aligned to the genome and two containing the unaligned genes or those aligning with unassembled contigs. The categories with aligned genes are illustrated schematically in Figure 2. The "potential ortholog" category (Figure 2a) included all the sequences that fully or partially overlapped exons of known genes on the genome, and which might represent orthologs missed by the Ensembl pipeline. The genes that could be aligned to intergenic regions of the genome were included in the "new gene" category (Figure 2b) and might be considered a source for putative new species-specific genes identified in the genome. The "gene variant" category (Figure 2c) included all the sequences that overlapped known exons but also aligned to unannotated or intronic regions that may indicate immature transcripts, transcript variants or different gene structures. Finally, genes that were completely aligned within one or more introns of a known gene were classified as "intronic" (Figure 2d) and probably constitute nested genes. All the genes not producing a significant alignment with the genome or aligned to unassembled contigs, where the annotation was not available, were classified respectively as "not aligned" and "contig".

**Figure 3:**
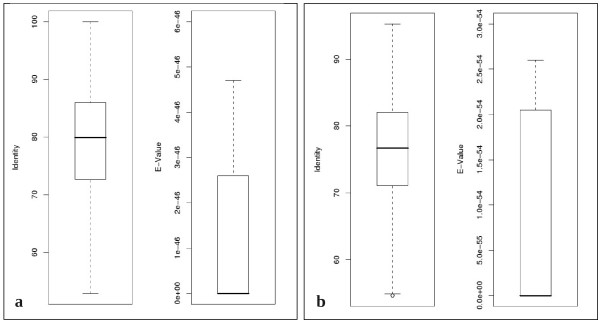
**Statistical box plot representing the distribution of identity percentage and E-value of alignments in "cow *vs*. human" (a) and "human *vs*. cow" (b) comparisons**.

The 3,801 bovine genes obtained from the "cow *vs*. human" comparison, were aligned to the human genome: 3,390 (89%) were mapped with a variable degrees of identity, while 411 (11%) sequences had no hit on the human genome. The statistical box plots in Figure 3a show that the median percentage identity of the best hits was about 80%, while the lowest value was around 53%, with an E-value lower than 5e-46, indicating highly significant alignments. Table 1 (column "cow *vs*. human") shows that 2,533 sequences (67%) were identified that overlapped known genes on the human genome, 472 (12%) fell into the class "new gene" and 289 (8%) were classified as "gene variant".

**Table 1:** Classification of bovine and human non-orthologous genes in categories according to the alignment results

	cow *vs*. human		human *vs*. cow	
	
	n. of genes	% of the total	n. of genes	% of the total
aligned genes	3,390	89	967	96
potential ortholog	2,533	67	120	12
new gene	472	12	500	50
gene variant	289	8	294	29
intronic	96	2	43	4
contig	-	-	10	1
not aligned	411	11	43	4
total non-orthologous genes	3,801	-	1,010	-

The same procedure was carried out for the "human *vs*. cow" comparison; 967 (96%) human sequences out of 1,010 were mapped to the bovine genome. While 43 (4%) had no hit at all, the statistical box plots in Figure 3b show that the median percentage of identity for the best hit of the mapped sequences on the genome is about 77% and does not fall below 55%, while the E-value is always below 2.6e-54. The aligned sequences were divided into the six categories described above (Table 1, column "human *vs*. cow"); 120 sequences could be aligned to known bovine genes ("potential ortholog" category). A considerable number of sequences fell into "new gene" and "gene variant" groups (500 and 294, respectively).

### Analysis of alignments

**Figure 4:**
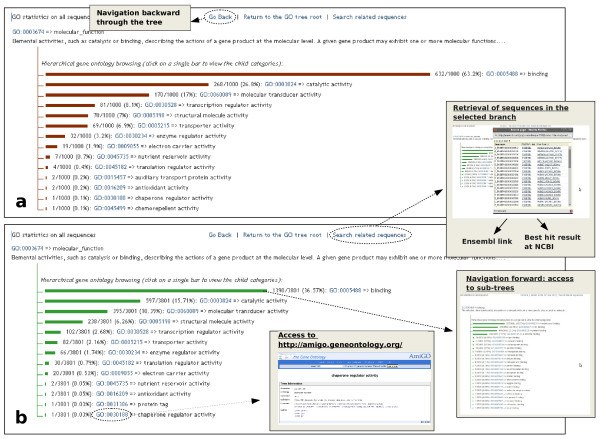
**Tool to display tree-like representation of the GO graphs**. Main root for molecular function category is shown for human (a) and bovine (b) libraries. The main navigation features of the web site are indicated by grey boxes.

All the bovine and human genes were classified by Gene Ontology (GO) terms and the GO tree was displayed with the web tool described in the gene ontology analysis section of the methods (Figure 4, [37]). The tree for the three main roots (molecular function, biological process and cellular component) was built for the whole set of sequences and for each of the five categories in which the sequences were classified according to the alignment results. Using this web tool the user can navigate through the tree and for each level can retrieve all the sequences described by a specific GO term. In addition, the tool has a direct link to the Ensembl gene viewer and, for bovine sequences, a link to the BLAST best protein hit at NCBI.

In cow, few GO terms were available for the bovine gene set and, even though a similarity search approach was used to provide new terms, about 36% (1,382) of the sequences, mainly from the "potential ortholog" (918) and "new gene" (310) libraries, could not be included in the tree. The majority of the sequences in the molecular function root were described as proteins with binding properties for other proteins, ions and nucleic acids. Other consistent classes contained genes involved in "catalytic activities" such as hydrolases, transferases and oxidoreductases or signal transducers.

A GO term could not be retrieved for about 10% (96) of human genes, mainly (67) coming from the "new gene" library. The distribution of the sequences in the human trees resembled those observed in cow with minor differences.

The biotype classification in Ensembl described all the 1,010 human genes as protein coding genes. However, among the 3,801 genes constituting the bovine libraries, a large number (1,114) was represented by different species of RNA (miRNA, rRNA, snRNA and snoRNA), while those remaining were annotated as protein coding (2,001), pseudogene (559) and retrotransposed genes (127). About 86% (1,728) of the protein coding genes could be aligned to the human genome.

As expected by the different level of annotation between the two species, 66% of the bovine protein coding genes are classified as *novel *while from human only 3.5% of the genes were novel (Table 2).

**Table 2:** Status of protein coding genes from the two comparisons

	cow *vs*. human		human *vs*. cow	
	
	known	novel	known	novel
potential orthologs	416	705	114	6
new genes	70	227	480	20
gene variant	129	131	289	5
intronic	8	42	42	1
not aligned	58	215	40	3
total	681	1,320	965	35

### Manual curation

**Figure 5:**
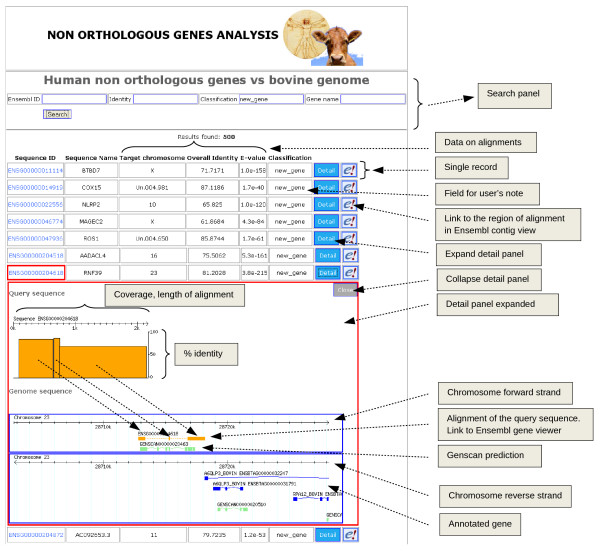
**Web interface to display the results of alignments of the bovine and human libraries**. Records can be retrieved by Ensembl ID, percentage of identity of the alignment, classification and gene name, through the search panel. For each record, representing a sequence, the Ensembl ID, the sequence name, the chromosome of the alignment, the percentage of identity, the E-value of the alignment and the classification flag were available. Moreover a link takes the user to the corresponding region of the alignment in the Ensembl contig view and the "detail" button expands a detail panel. In this panel the selected alignment is represented graphically in order to show the coverage and percentage of identity of the sequence but also the position of the alignment to the genome scaffold, alongside with *genscan *predictions and known gene sequences, retrieved directly from Ensembl database.

The accuracy of the classification generated by the pipeline was manually verified for all the categories, except "contig", for which the annotation information of genomic regions was not available. This process was performed using a web interface tool described in the alignments procedures section of the methods, where the data on the alignments are reported and graphically displayed (Figure 5, [38]). The web interface tool provides a direct external link to the Ensembl website, to allow all the information for a given gene to be easily accessed. Criteria used to evaluate the significance of the alignments, and therefore the correctness of the classification, were: the conservation of gene structure between human and cow, the parameters of alignments and the presence of *genscan *predictions, ESTs, mRNAs and UniGene features, aligned alongside with the query sequence. Only protein coding genes aligned to the genome with overall identity equal or greater than 75% were selected for the manual curation, even though the sequences below this threshold had highly significant E-values. The genes in the "not aligned" category, which could not be selected using a threshold, were all examined. The analyses and supporting evidence for each library is presented in the following sections and the results summarised in Table 3.

**Table 3:** Results of manual curation.

	cow *vs*. human				human *vs*. cow			
	
	potential orthologs	gene variants	new genes	intronic	potential orthologs	gene variants	new genes	intronic
pseudogenes and retrotransposed	17							

potential orthologs	6		2		13			

different length and structure	142	103			32			

additional exons		20						

unreliable	12	31		3	4	11	105	8

overlap Genscan or EST, potential new genes			64	13				

no evidence, potential new genes			46					

overlap different genes					16			

gene variants						144		

problems in annotation						25	2	

new genes/partial							125/55	

nested genes								11

examined (>75% identity)	177(883)	154	112	15	65	180	287	19
total	1121	260	297	60	120	500	294	43

Number of genes for each category are reported.

### *In silico *bovine Libraries

#### Potential Orthologs

This category had the largest number of genes with at least 75% of identity, almost 79% of the total, and contains 883 sequences aligned to exons of known human genes. Those with at least 90% identity (177) were systematically examined, however, additional random checks were performed on the whole library. In most of the cases (80%), bovine sequences were found to be shorter than the corresponding human genes, with bovine transcripts clearly missing one or more exons at the gene boundaries. In addition, for some short cow genes the alignments showed a relationship with nearby genes, suggesting that they might be wrongly annotated as distinct genes, while in fact they represent a single gene. Short genes were also represented by terminal exons of misassembled genes which also showed matches elsewhere in the genome. Many of these short genes have been removed in recent releases of Ensembl (51 and 52 in particular). The comparison between cow and human genomes through these alignments, showed that many cow genes had additional evidence aligned alongside the corresponding gene region(s), such as ESTs, mRNAs and *genscans *to support the presence of additional exons, which had not been annotated, in the proximity of the bovine genes. In a few cases (10%), bovine sequences could be aligned with human pseudogenes or retrotransposed genes. Potentially, real orthologs were identified only for short sequences and other particular situations, such as genes located at the ends of contigs or on unassigned chromosomes that appeared truncated or with wrongly assembled structure. Artefacts (i.e. non informative alignments due to the high percentage of identity but very low coverage of the sequence) and nested genes constituted a minority of this class.

#### Gene Variants

The total number of sequences examined in this class was 154, and for 67% substantial differences in gene length and structure were observed between cow and human genes. These sequences probably represent species-specific differences in gene architecture. In 13% of the sequences the presence of additional exons is suggested in the structure of the human gene, which was confirmed by the quality of the alignments and the presence of *genscan *predictions. The remaining 20% of the results showed unreliable alignments and are likely to be alignment artefacts resulting from short sequences or short stretched of high homology in otherwise divergent sequence.

#### New Genes

The bovine sequences aligned in intergenic regions of the human genome were classified as possible new human genes; of these 112 sequences were examined in detail. Results for 57% of these annotated genes showed an overlap with *genscan *predictions and EST features that aligned with the same regions of the human genome. Many of these features showed good correspondence with the structure of the bovine gene to which they aligned. The results showed that 41% of the genes aligned with empty regions of the genome where no other annotated features, such as ESTs or *genscans*, were present. The remaining 2% of the sequences were re-classified as potential orthologs as they were annotated as genes in later releases of Ensembl (51 and 52). In particular new human genes were annotated in these later releases at the same position where the pipeline described in this paper had placed the corresponding bovine gene.

#### Intronic

Sixteen cow sequences aligned completely within introns of annotated human genes. Of these sequences, 81% showed an overlap with existing *genscan *or ESTs features aligned in the same region of the human genome. Overall 19% of the results showed unreliable alignments.

#### Not Aligned

Among the bovine sequences that did not align to the human genome, 273 were protein coding, most (79%) were annotated as "novel genes" by Ensembl (Table 2). When aligned with BLASTX against the protein non-redundant database at NCBI, 11% had a hit with human sequences, BAC clones and synthetic constructs (e.g. plasmids, clones) and 23% had a match with an E-value lower than 1e-10 with sequences from other species, such as sheep and goat. In addition, 29% showed similarity to the nucleotide database, both with human and bovine sequences, while the remaining 37% had no match at all. The problem of finding a significant match within this library is partly due to 25% of the sequences being less than 100 nucleotides in length, and therefore most are likely to be artefacts, short open reading frames or fragments of sequences, indeed 33% of them were located on unassigned chromosomes. Ensembl reported no evidence (*genscan *models, ESTs, UniGene features) for 52% of these sequences while significant evidence was available only for 12%. The remaining sequences had only partial and unreliable evidence. Some of the sequences showing a good gene structure, but that originally had no match with the protein database, have had protein evidence added in a more recent Ensembl release (version 53). Within this last group only one gene is described as "known", with good supporting evidence and is annotated as "Stella Fragment" (Developmental pluripotency-associated protein 3, DPPA3; ENSBTAG00000038326). The same gene is present in human and mouse but is not classified as orthologous in cow. Even though the annotated bovine gene appears to be a fragment, the sequence diverges significantly from the sequence of the other species.

### *In silico *human libraries

#### Potential Orthologs

- Human genes aligned to the bovine genome, and having an overlap only with known exons of annotated genes, were classified as "potential orthologs". In total 65 genes were examined and fell into four different classification groups. Despite the good overlap with annotated bovine exons, 50% of these sequences were either shorter or longer than the corresponding bovine gene, i.e. had either different number of exons or exons with different length. These genes were most likely not annotated as orthologs because, in the current sequence assemblies, they have species-specific features. In the second case, 24% of the sequences overlapped two different bovine genes located in the same chromosomal region, or novel bovine genes with a single exon. The presence of bovine *genscan *predictions and other EST features in the same region suggested annotation or sequence assembly problems with the bovine genome in that particular position. Of the sequences missed by the Ensembl automated annotation pipeline, 20% could be considered as orthologs as they have a good structure and sequence overlap with bovine genes. The remaining 6% of the sequences showed a non-reliable alignment (artefacts) or aligned to pseudogenes.

#### Gene Variants

Human genes aligned to bovine genome and having an overlap both with exons and introns or upstream/downstream regions of known bovine genes, were classified as "gene variants". Several different examples were identified within the 180 genes examined. The alignment of human gene sequences with the presence of *genscan *predictions or ESTs features together with the quality of the match, confirmed 80% of the sequences as potential "gene variants". For 14% of the sequences, problems with the annotation were observed, e.g. where a human gene matched with two different bovine genes in the same region; these are probably bovine genes placed on misassembled regions of the genome sequence, or that had been incorrectly annotated as two distinct genes. The remaining 6% of the results were considered unreliable alignments (i.e. short alignments or sequencing artefacts).

#### New Genes

Human sequences aligned to intergenic regions, i.e. where no genes had been identified, were classified as "new genes". This category had 287 protein coding genes of which 44% could be considered new bovine genes. These findings were also supported by new information introduced in the latest releases of Ensembl (from 51 onward) where, for the 26% of those sequences verified as potential "new gene" by the manual curation, a new Ensembl feature identified as "EST based gene" was found in the same position corresponding as produced by our pipeline. For 19% of the "new genes" aligned to genomic regions with supporting evidence such as *genscan *predictions, ESTs, or mRNAs, the evidence and overlap were incomplete and therefore these sequences were classified as "partial new genes". Similar situations were observed where human genes overlapped two or more *genscan *predictions, or aligned within very large predicted genes, which suggested problems in the annotation of specific bovine genome regions. The remaining results contained short sequences, fragments and artifacts that produced unreliable alignments. In some cases, human genes aligned with *genscan *exons that were spaced at large distances on the bovine scaffold, or had specific parts of the gene matching with different regions of the genome. A particular example of this situation was found for olfactory receptors, which are recurrent motifs in the dataset of sequences. These probably represent domains or repeated gene structures showing high similarity with sequences at many different chromosomal regions.

#### Intronic

This category includes all the human sequences aligned completely within introns of known bovine genes. A total of 19 sequences were examined and 57%, were considered possible new nested genes. The remaining 42% of the results represented artefacts or short aligned sequences (less than 100-150 nucleotides in length).

#### Not Aligned

Overall 43 human sequences had no match with the bovine genome. Forty sequences were *known *genes and three were *novel *genes (Table 2). These sequences were translated in all six reading frames and BLASTed against the protein non-redundant database: for 35 a match was found with a bovine sequence and in the 29% of the cases the annotation and description corresponded to that for the human gene.

## Discussion

Over the last decade, many different approaches for identifying gene orthology between species have been proposed in the literature. The process of gene annotation, as well as the discrimination between protein coding and non coding genes [39], will become even more important as the number of available genome sequences increases, in line with the rapid progress of the sequencing technology. Depending on the sensitivity and specificity of methods used to identify orthologous genes, the fraction of genes without orthologs between species is variable, and also depends on the quality of the genome assembly [40,41]. Among these genes, there are the so called orphans [32], which have no homologs among the genes of other species. Even though several explanations have been proposed for the absence of homologs, one of the possibilities is that they might represent species specific genes. In the literature, the search for orphans genes has been carried out in different species by comparing gene sets at protein level [34,42,43]. In the work presented here we faced the problem of non-orthologous genes between species at nucleotide level. We focused on the bovine genome (version 4.0), whose assembly and annotation is still ongoing. Ensembl orthology predictions from release 50 were used, as these represented the highest quality genome annotations across several mammalian species. Ensembl automatically produces orthology predictions between species and for each release of the database these predictions can be easily queried using the "BioMart" tool. A simple query to obtain the number of non-orthologous genes between the bovine and the human genome returned 5,507 out of 22,836 genes. The reverse query returned 19,811 out of 36,396 human genes with no orthologs in the bovine genome. The differences are dependent on the level and quality of annotation for the two genomes, and on the larger set of annotated human genes. In order to reduce this effect in a simple two-way comparison, the bovine and human datasets were filtered with information coming from other completed genomes, specifically mouse and dog. A total of 3,801 bovine genes had no orthologs with these three species while 1,010 human genes, with orthologs in mouse and dog, had no orthologs in cow (Figure 1). These two groups of genes were considered as the most consistent non-orthologous genes to use in further work. In the previous assembly of the cow genome (Btau 3.1, Ensembl release 49), a similar query gave almost double the number of bovine non-orthologous genes (6,247), while non-orthologous human genes were slightly fewer (865). This reflects major improvements in the bovine genome assembly and annotation between version 3.1 and 4.0, but suggests that there are still problems either with the assembly or the annotation of the bovine sequence.

The two sets of non-orthologous genes (cow *vs*. human and human *vs*. cow) were investigated in order to test the quality of orthology predictions, to reveal genuine differences between species and most commonly show problems with the genome assemblies. A bioinformatic pipeline and web tool were developed to describe the alignments of each library with the genome of the other species, and the alignments were classified into 5 different categories, according to the annotation associated with the sequence in each genome (Figure 2, Table 1). These classes were established according to the different scenarios that might explain annotation problems, which were: potential orthologs, gene variants, new genes, intronic genes and not-aligned sequences. For this analysis only the protein coding genes were selected, which most likely represent functional genes, while pseudogenes and retrotransposed genes were removed as the non-coding RNAs, which were analysed separately [44].

Although all the aligned sequences showed highly significant E-values, only results with more than 75% of overall identity were targeted for a detailed manual curation. A web based informatic tool was created and used that provides easy access to the alignments and available annotation for each gene.

Among the genes examined, 90% of the sequences had a significant match, even though for a small fraction the alignments were not reliable. These included very short sequences and genes which had short alignments or that aligned with two different genes within the same genomic region, and were considered sequence or alignment artefacts. These "problematic" sequences were distributed throughout the genome and did not suggest the presence of localised regions with problems with the genome assembly or annotation (data not shown).

The current level of annotation of the bovine genome is not comparable with that of human, however the alignment of the annotated bovine genes with the human genome produced some interesting results. In some cases there was evidence to suggest new, presently unannotated, features in the human genome, including additional exons, as observed in the "gene variant" class, or potential new human genes, from the "new gene" and "intronic" classes. The latter were supported by the presence of other evidence in the region of the alignments, such as the coincident alignment of EST and *genscan *predictions. Indeed, some of the features identified appeared in later releases of Ensembl database, where additional human genes have been annotated exactly where the pipeline used here had aligned a bovine gene. This observation supports the value of this type of comparative approach. The "potential ortholog" class helped to identify additional orthology relationships, however, it also identified deficiencies in the genome sequence and errors in the annotation of many bovine genes. Generally, the annotation suggested that cow genes were shorter than the human orthologous genes, which in many cases was because exons had been missed at gene boundaries. Alignment of EST and *genscan *predictions, in the corresponding positions of the bovine genome, suggested the presence of new bovine exons. In addition many genes were identified in the bovine genome that had not been annotated.

It would be expected that genes with orthologs in human, mouse and dog should have homology relationships in cow, even though they had not been identified by the automated orthology prediction. Thus, the alignment of the human genes to the bovine genome should find new features to improve genome annotation in cow. From the results in the "new gene" class, 46% could be considered as new bovine genes, indeed in latest Ensembl releases half of those identified using the approach described here were added in a new Ensembl feature called "EST based genes", which were in agreement with our alignments. The interpretation of the results for genes in the "potential ortholog", "gene variant" and "intronic" classes, becomes more complex as it is not completely clear if the observed alignments and differences are due to species-specific features, or problems with the bovine annotation or the genome assembly. From the genes belonging to "potential orthologs", 20% may be considered as true orthologs which were missed by the Ensembl prediction pipeline, for the most part due to minor differences between the sequences. Accepting the current annotation of the bovine genome, 80% of the results in the "gene variant" class were highlighted the presence of new exons for genes currently annotated in cow.

The "not aligned" class may contain real non-orthologs between the four species but also orphan genes with no match with other species. This class was analysed for both cow and human genomes, by searching similarities with the complete non-redundant protein database from NCBI. For most of the human sequences, a match was identified with bovine proteins whose annotation and description is exactly the same as in human. These results most probably represent gene sequences that are still not annotated or assembled into the bovine genome, and hence were completely missed by the Ensembl orthology prediction. Some of the cow genes for which there is no match with the human genome may be indeed *novel*, bovine orphan genes, as only 11% in this class had a significant match with a human sequence and 37% had no match at all in the NCBI database. Among these genes there are novel sequences which also have supporting protein evidence; these are interesting candidates among which to look for cow specific coding regions. The functions of orphan genes are generally poorly characterized [43], they show distinctive features such as high tissue specificity, rapid evolution and short peptide size [34]. Recent works have demonstrated that they evolve three to four times faster than the average genes in Drosophila [43] and in primates [34]. In some cases the sequence divergence between species may be so great that the orthology between the genes is not obvious. This situation is represented by the "Stella fragment" related gene (DPPE3), which is annotated and has good supporting evidence. Indeed this gene has human and mouse counterparts but with the sequences highly divergent between the species.

The discrimination between orthologs and paralogs still remains difficult, especially when comparing incomplete and large genomes, as addressed by Fulton et al. [45]. Genes predicted as paralogs by Ensembl are 49% and 60% of the bovine and human libraries, respectively. Paralogs, which mainly arise from a duplication event and may undergo structural rearrangements during evolution [1], are found in the non-orthologous sets described herein. Their sequence divergence might explain why they were missed as orthologs between species and in some cases can be traced back with the similarity approach used in this work.

Ontology descriptions, even if not complete for the bovine gene set, due to the lower level of annotation, were interesting in describing the groups of genes created in this work. Many of the genes with no apparent orthologs were clustered as proteins with binding properties. The typical modular composition of such proteins and their specificity for different ligands could explain structural differences which might have an effect on the orthology prediction. Despite the annotation and similarity search performed to retrieve GO terms for the bovine non orthologous genes, no valid annotation was found for the 75% of the cow genes in the "not aligned" group. This highlights the need to focus on this particular group of genes that might reveal orphan as well as species specific coding sequences.

## Conclusions

This study was focused on a particular class of genes predicted to be non-orthologous between cow and human genomes. These genes are normally considered the result of divergent evolution and are reported by the automated pipelines and following the manual annotations of the bovine and dog genome in the international sequencing projects [31,36]. Evidence found within this work suggest that a high number of non-orthologous genes between cow and human could be considered a side effect of an incomplete genome assembly and annotation process. The approach described here allowed the differences and similarities for this class of genes to be highlighted and also possible new features for the human genome to be identified. The comparison between species, using similarity and classification methods, is crucial for the analysis of genome sequences and gene sets, especially when the annotation process relies on the quantity and quality of available data on transcript sequences for a particular species. In this context, this kind of comparative approach could be used to extend the current genome annotation protocols. The presence of non-orthologous genes in other species should be considered as a central resource to derive important information for the definition of gene models and structures, in particular for the newly assembled genomes, where the lack of a complete set of genes and poor transcript sequences information may restrict the commonly applied annotation procedures.

## Methods

### Datasets

The cow genome assembly version 4.0 and the human genome assembly version 36 were downloaded from Ensembl release 50 [46]. Gene sets were retrieved directly from Ensembl database using the multi-species comparison options in the BioMart web interface [47]. In detail, the bovine library of non-orthologous genes was obtained by retrieving three sets of Gene IDs obtained after applying a filter to exclude the orthologous genes of human, mouse and dog, respectively 5,507, 5,273 and 5,842 genes. Only the genes in common among these three sets (3,801) were retained (Figure 1a). The second library is referred as "human"; this refers to the human subset of genes that are found in common between human, mouse and dog genomes and represent core mammalian orthologs. Two lists of human genes were built; the first contained orthologs between human and mouse datasets filtered to exclude the orthologous genes of cow and the second by the orthologs between human and dog datasets, filtered to exclude the orthologous genes of cow. Only human Gene IDs in common between these two lists (respectively 2,104 and 1,437 genes) were retained for a total of 1,010 genes (Figure 1b). All the possible orthology predictions (1:1, many:1, 1:many, many:many) as provided by Ensembl were used.

The protein and nucleotide non-redundant databases were downloaded from NCBI on January 2009 [48].

### Statistical plots

Statistical box plots and distributions were drawn using R v2.7.0 [49]. In this analysis, the best hit with the lowest E-value for each sequence was considered.

### Alignment procedure

All the alignments were done using WU-BLAST v2.0 [50], with identity matrix for nucleotide alignments and BLOSUM62 identity matrix for protein alignments. The longest transcript (canonical transcript) for each gene was aligned to the whole genome sequence using the parameters W = 10, E = 1e-5, links, topcomboN = 1 and hspsepSmax = 1,000,000. The best hit with the lowest E-value was considered as the result for each alignment. Custom Perl scripts were written to run the alignments, parse the results using BioPerl v1.5.2 libraries [51] and store the information into a relational MySQL v5.0 database [52]. The results were classified according to the genomic position and to the current annotation available for each species, using the Ensembl Perl API to query the Ensembl database and retrieve all the genomic information [27,53]. A web interface was created to query the database of results and display the aligned sequences and the corresponding genomic regions, with other annotated genes and the *genscan *[54] predictions available. This interface was written using the Ruby language and was based on the Ruby on Rails framework [55]. The interface was used to perform the manual annotation and visual inspection of the results, providing the possibility to query the database of results directly using different parameters, such as alignment identities, gene IDs and classification labels. Direct access to the Ensembl information for each gene was added to help the retrieval of all the necessary information during the manual annotation phase. The interface is available at http://www.itb.cnr.it/idralab/non_orthologs.

### Gene Ontology (GO) Analysis

We obtained Human GO annotations via the Biomart searching tool from Ensembl release 52 [46,47]. The Gene Ontology Database (January, 2009)[56,57] was used to extend this preliminary GO data set with all the "is-a" related GO terms (e.g. GO:0004601 - peroxidase activity - "is a" GO:00016209 - antioxidant activity), up to the three main roots (molecular function, cellular component and biological process) in the Gene Ontology hierarchy.

Bovine GO annotations derive from the EBI Gene Ontology Annotation (GOA) database (January, 2009) [58,59], without distinguishing between the available evidence codes. In details, sequences were blasted (BLASTX, e-value threshold of 1e-10) against a modified version of the UniprotKB database (January, 2009)[60,61] containing only GOA annotated proteins, and were linked to GO annotations in accordance with the GOA database. As in the case of human sequences, "is-a" related GO terms were added, according to the GO DAG available data.

Perl scripts were designed to produce library-specific statistics on the sequence distribution among the GO terms, and to save the data into a relational MySQL v 5.0 database [52].

A software tool written in PHP language was developed in order to display the sequence-GO distribution. This visualization tool dynamically creates a navigable, tree-like representation of the GO graph, showing each GO term as a bar proportionally long as the number of the related sequences (Figure 4). The visualisation tool is available at http://www.itb.cnr.it/ptp/annotation.

## Authors' contributions

RM and FS carried out the bioinformatic analyses and wrote the paper; AC performed the GO analysis; PAM and JLW critically reviewed the paper. All the authors read and approved the final manuscript.
